# Supplementary material for: Seminoma and Embryonal Carcinoma Footprints Identified by Analysis of Integrated Genome-Wide Epigenetic and Expression Profiles of Germ Cell Cancer Cell Lines
Source: PLoS One. 2014 Jun 2;9(6):e98330. doi: 10.1371/journal.pone.0098330 (PMC4041891; doi:10.1371/journal.pone.0098330)
Supplement: File S1 — ZIP file containing DMRforPairs output for significant regions. Please start from the html files. (ZIP) [file pone.0098330.s008.zip › figures/34149.pdf]

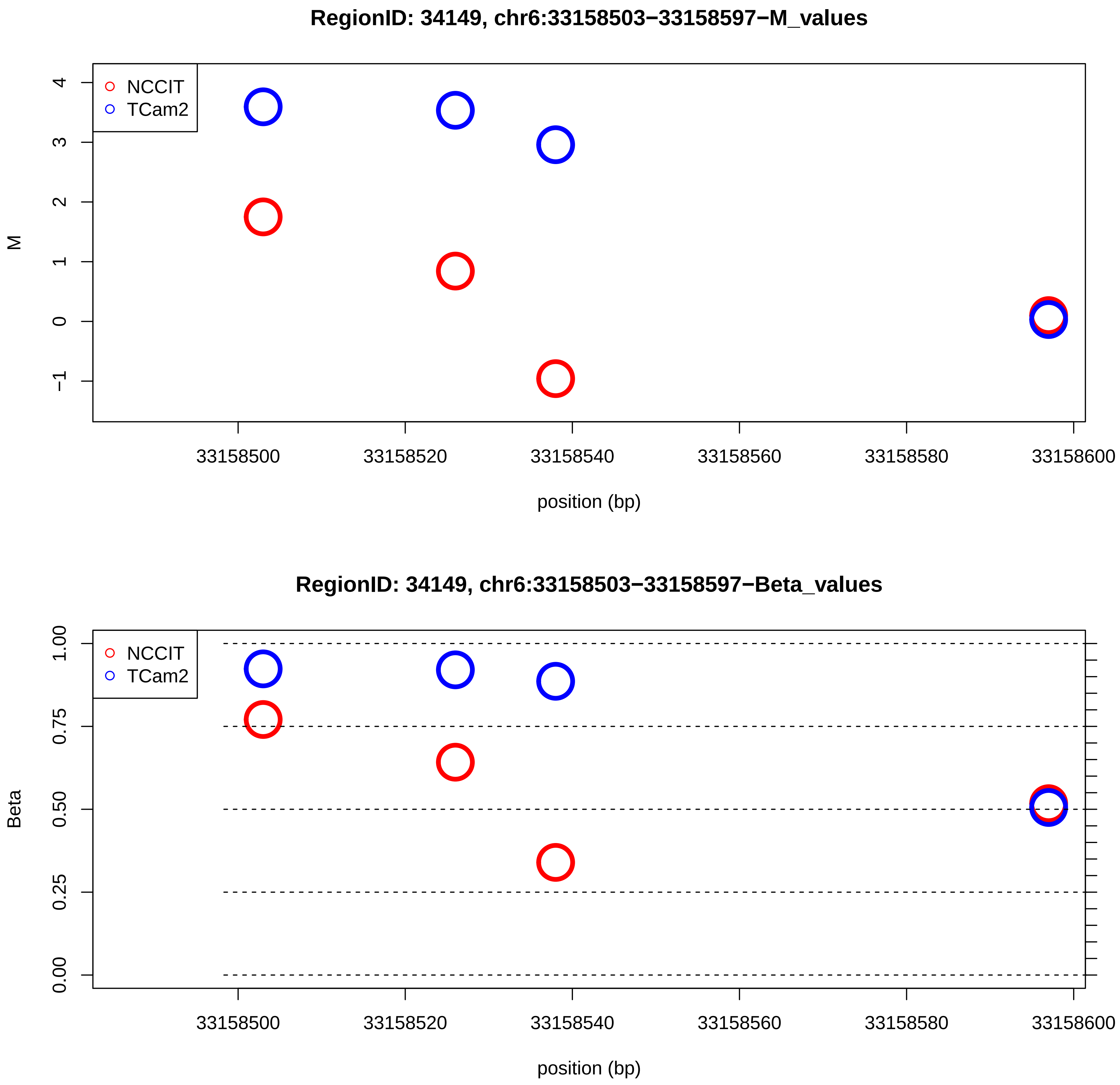
RegionID: 34149, chr6:33158503−33158597−M_values
NCCIT
TCam2
M
4
3
2
1
0
−1
33158500
33158520
33158540
33158560
33158580
33158600
position (bp)
RegionID: 34149, chr6:33158503−33158597−Beta_values
NCCIT
TCam2
Beta
1.00
0.75
0.50
0.25
0.00
33158500
33158520
33158540
33158560
33158580
33158600
position (bp)
